# Nanoarchitectonics of highly dispersed polythiophene on paper for accurate quantitative detection of metal ions†

**DOI:** 10.1039/d3ra08429a

**Published:** 2024-02-08

**Authors:** Yui Sasaki, Xiaojun Lyu, Takayuki Kawashima, Yijing Zhang, Kohei Ohshiro, Kiyosumi Okabe, Kazuhiko Tsuchiya, Tsuyoshi Minami

**Affiliations:** a Institute of Industrial Science, The University of Tokyo 4-6-1 Komaba, Meguro-ku Tokyo 153-8505 Japan tminami@g.ecc.u-tokyo.ac.jp; b JST, PRESTO 4-1-8 Honcho Kawaguchi Saitama 332-0012 Japan

## Abstract

π-Conjugated polymers such as polythiophene provide intramolecular wire effects upon analyte capture, which contribute to sensitive detection in chemical sensing. However, inherent aggregation-induced quenching causes difficulty in fluorescent chemical sensing in the solid state. Herein, we propose a solid-state fluorescent chemosensor array device made of a paper substrate (PCSAD) for the qualitative and quantitative detection of metal ions. A polythiophene derivative modified by dipicolylamine moieties (1_poly_), which shows optical changes upon the addition of target metal ions (*i.e.*, Cu^2+^, Cd^2+^, Ni^2+^, Co^2+^, Pb^2+^, Zn^2+^, and Hg^2+^), was highly dispersed on the paper substrate using office apparatus. In this regard, morphological observation of the PCSAD after printing of 1_poly_ suggested the contribution of the fiber structures of the paper substrate to the homogeneous dispersion of 1_poly_ ink to suppress aggregation-induced quenching. The optical changes in the PCSAD upon the addition of metal ions was rapidly recorded using a smartphone, which was further applied to imaging analysis and pattern recognition techniques for high-throughput sensing. Indeed, the printed PCSAD embedded with 1_poly_ achieved the accurate detection of metal ions at ppm levels contained in river water. The limit of detection of the PCSAD-based sensing system using a smartphone (48 ppb for Cu^2+^ ions) is comparable to that of a solution-based sensing system using a stationary spectrophotometer (16 ppb for Cu^2+^ ions). Therefore, the methodology based on a combination of a paper-based sensor array and a π-conjugated polymer will be a promising approach for solid-state fluorescent chemosensors.

## Abstract 2

Paper fibers allow the high dispersion of a fluorescent polythiophene chemosensor for the suppression of aggregation-induced quenching, which is applied to the solid-state fluorescent quantitative sensing of metal ions using imaging analysis and pattern recognition techniques.

## Graphical abstract

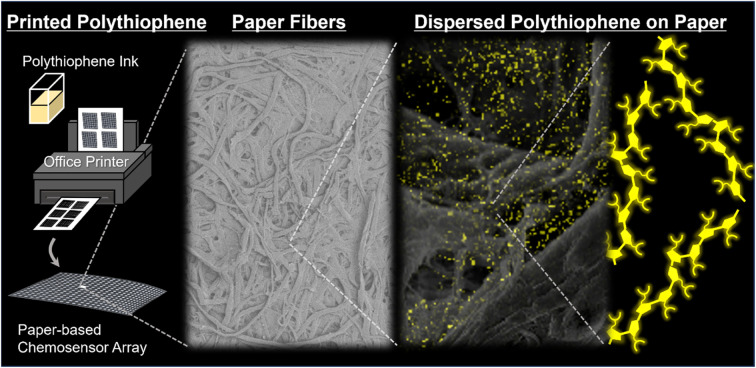

## Introduction

The development of portable assessment methods is in high demand, with an increase in the attention given to environmental protection.^1^ In this regard, invisible pollutants such as heavy metal ions in environmental water cause damage to organs in the immune system, stemming from the intake of food contaminated with heavy metal ions.^2^ In particular, a combination of metal ions (*e.g.*, nickel(ii) (Ni^2+^), cobalt(ii) (Co^2+^), and copper(ii) (Cu^2+^) ions) has an unexpected influence on biological systems, such as inhibition of the growth of green algae^3^ and the generation of metal-ion-resistant microbes.^4^ Given that the monitoring of metal ion levels in environmental media is significant, the development of easy-to-use devices is required for the accurate analysis of metal ions. Conventionally, large instrumental apparatus (*e.g.*, inductively coupled plasma mass spectrometry (ICP-MS),^5^ atomic absorption spectrometry (AAS),^6^ and atomic fluorescence spectrometry (AFS)^7^) have been applied to assess metal ion levels, but the feasibility of on-site sensing using them is a concern because of size limitations and their complicated detection mechanisms. Hence, approaches using small-sized sensor devices are required for on-site analysis in practical sensing situations.

Paper is an attractive material for portable and disposable chemical sensors owing to its beneficial properties of eco-friendliness, high water-absorbability, and capillary ability.^8–10^ For example, pH test strips and urine analysis kits made of paper materials have already been used in real-world scenarios. However, conventional paper-based sensor devices enable only qualitative analysis because the sensing mechanisms are simplified for naked-eye detection, which indicates the difficulty of simultaneous quantitative detection of various analytes at different concentrations.^11^ Meanwhile, the concept of sensor arrays inspired by the mammalian olfactory system allows the simultaneous detection of various analytes, based on a combination of cross-reactive receptors and powerful data-processing methods.^12–14^ In these array designs, the selection of cross-reactive receptors and transducer units significantly affects the quality of inset data for pattern recognition. In contrast to biological materials (*i.e.*, enzymes and antibodies) with highly selective recognition abilities derived from the ideal lock-and-key principle, artificial receptors are superior to cross-reactive recognition because of their moderate binding affinities to similar structural analytes.^15,16^ As transducer units, optical responses in chemical sensing are easily recognized and recorded without using any large instrument. Thus, we decided to focus on chemosensors comprising artificial receptors and optical reporters for high-throughput analysis using a paper device. Chemosensors are capable of visualizing analyte-capture information as colorimetric and/or fluorescence responses in chemical sensing.^17^ Although chemosensors and their arrays for metal ions have been vigorously developed in supramolecular chemistry fields,^18–24^ the realization of paper-based chemosensor array devices (PCSADs) for quantitative detection is still challenging. For example, fluorescent chemosensors allow higher sensitivity than colorimetric chemosensors, but aggregation-induced quenching cannot be ignored in solid-state fluorescent chemosensors.^25^ Fabrication technology is significant for obtaining reproducible sensing results in solid-state fluorescent chemosensors. However, the methodology for device fabrication and data analysis has not yet been satisfactorily established. Therefore, this study aims to establish a methodology to avoid aggregation-induced quenching for the fabrication of a solid-state fluorescent chemosensor on a paper substrate.

**Fig. 1:**
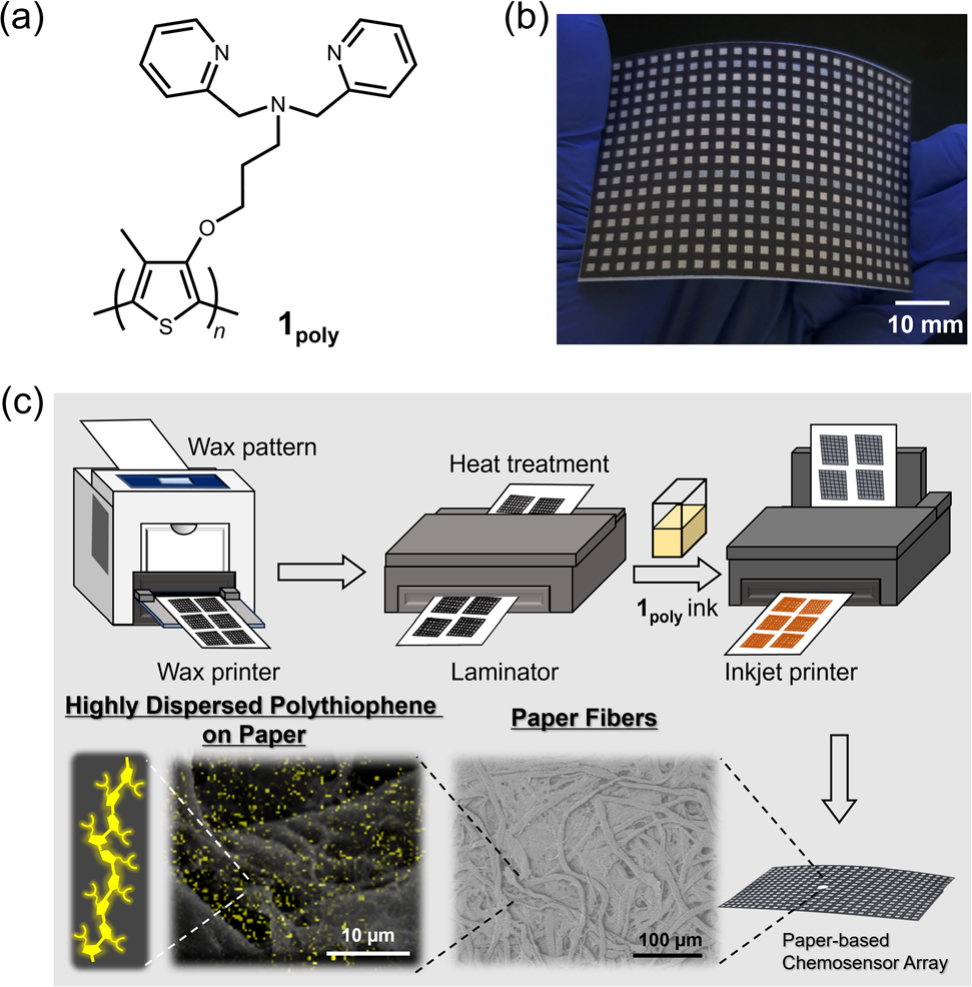
(a) Chemical structure of 1_poly_. (b) Photograph of the 384-well microtiter PCSAD embedded with 1_poly_. The printed PCSAD showed various optical responses upon the addition of metal ions. (c) Conceptual figure representing effects of paper fibers on the dispersion of 1_poly_ by printing methods. The surface morphology of the PCSAD embedded with 1_poly_ was characterized by SEM observations with different magnifications. Yellow dots observed on the paper fibers indicate an elemental component of 1_poly_ originating from sulfur atoms in the EDX-mapping image.

Polythiophenes (PTs) are representative organic materials showing intramolecular wire effects in chemical sensing, and their beneficial properties have been widely applied to fluorescent chemosensors.^26–31^ PT-based fluorescent chemosensors cause self-aggregation quenching in the solid state, resulting in the suppression of intramolecular wire effects.^31^ Therefore, strategies such as dispersion^31–33^ or isolation^34,35^ of polymer wires are required to inhibit self-aggregation for the development of solid-state fluorescent PT-based chemosensor devices. In this regard, fiber structures of paper materials can potentially be applied to disperse PT derivatives to avoid aggregation-induced quenching in solid-state chemosensor devices. However, the effect of paper fibers on the dispersion of π-conjugated polymers has not been evaluated in paper-based fluorescent chemosensor devices. For this purpose, an ON–OFF-type PT sensor was employed to evaluate the role of fiber structures of the paper substrate for the suppression of aggregation-induced quenching. In this assay, a dipicolylamine (dpa) moiety as a ligand for metal ions^36–38^ was introduced into the PT backbone for metal ion sensing. The dpa ligands with Lewis-base property were applied to artificial receptors for Lewis-acidic metal ions.^28,39^ The inherent cross-reactivities of the dpa-based receptors for metal ions potentially contribute to pattern recognition.^19,40^ The designed and synthesized chemosensor, a dipicolylamine (dpa)-attached PT (1_poly_) (Fig. 1(a)), was used as printing ink for the fabrication of a 384-well microtiter PCSAD (Fig. 1(b)). The polymer wires were highly dispersed on the array device which possessed fiber structures by using printing technology, which showed fluorescence owing to the homogeneous distribution of the polymer wires (Fig. 1(c)). The fluorescence responses on the PCSAD by adding target metal ions were rapidly captured using a smartphone, followed by imaging analysis and pattern recognition for high-throughput sensing. The applicability of the fluorescent PCSAD for real-sample analysis was evaluated by a quantitative assay in river water.

## Experimental section

### Materials

All reagents and samples were used without further purification. Methanol (MeOH) and hydrochloric acid (HCl) were obtained from Kanto Chemical Co., Inc. 2-Morpholinoethanesulfonic acid (MES) was purchased from Dojindo Laboratories Co., Ltd. Reagents purchased from Sigma-Aldrich Co. LLC. were zinc (Zn^2+^) perchlorate, copper (Cu^2+^) perchlorate, nickel (Ni^2+^) perchlorate, cobalt (Co^2+^) perchlorate, lead (Pb^2+^) perchlorate, and mercury (Hg^2+^) perchlorate. Sodium hydroxide (NaOH), a commercially available river sample with trace elements in river water (Elevated Level, NMIJ CRM 7202-c), and Daigo's artificial seawater SP for marine microalgae medium were obtained from FUJIFILM Wako Pure Chemical Industries, Ltd. The synthetic methodology for 1_poly_ was established in our previous report.^41^

### UV-vis and fluorescence measurements

The optical properties of 1_poly_ were evaluated in an aqueous MeOH solution (MeOH : water = 3 : 1, v/v) containing MES (12.5 mM) and NaCl (12.5 mM) of pH 5.5 at 25 °C. UV-vis absorption spectra of 1_poly_ (4.0 × 10^−4^ M per unit) were recorded using a Shimadzu UV-2600 spectrophotometer within the wavelength range 300 to 650 nm. A Hitachi F-7100 spectrophotometer was applied to record the fluorescence emission spectra of 1_poly_ (4.0 × 10^−5^ M per unit) from 450 to 750 nm at a scan rate of 240 nm min^−1^. The slit widths for excitation and emission were set to 20 and 10 nm, respectively. The excitation wavelength for metal ion detection was selected as 425 nm according to an isosbestic point observed in the UV-vis titrations of 1_poly_ for metal ions.

### ESI-MS analysis

The coordination of a thiophene monomer (1) and a metal ion was evaluated by electrospray ionization-mass spectrometry (ESI-MS) using a Shimadzu LC2010CHT and a Shimadzu LCMS-2020 controlled by Shimadzu LabSolutions. The ESI-MS was operated in scan mode with interface potential of +4.5 kV, flow rate of nebulizer N_2_ gas of 1.5 L min^−1^, capillary temperature of 350 °C, and Qarray RF voltage of 60 V. Milli-Q water was used as an eluent.

### Fabrication scheme for the 384-well microtiter PCSAD

The PCSADs were fabricated using commercially available office printers. An array pattern with a 384-microtiter plate (2 mm × 2 mm per well) for the printing process was designed using Microsoft PowerPoint 365. Filter paper adjusted to A4 size (Whatman Grade 1, GE Healthcare, UK) was employed as the paper substrate for the printed array devices. The surface of the filter paper was functionalized with a hydrophobic wax pattern using an office wax printer (ColorQube 8580N, Xerox). Meanwhile, the reverse of the filter paper was modified by a thin wax layer, followed by annealing treatment of the paper device using a hot plate apparatus (NHS-450ND, NISSIN Co., Japan) at 150 °C for 30 s. A lamination film (LZ-A4100, IRIS Ohyama, Japan) covered the reverse of the printed filter paper to enhance mechanical robustness. The paper substrate was annealed by using an office laminator (QHE325, Meikoshokai, Japan) at 150 °C for 20 s. A printing ink made of 1_poly_ (4.0 × 10^−4^ M per unit) in an aqueous MeOH solution (MeOH : water = 3 : 1, v/v) containing MES (1 mM) and NaCl (1 mM) of pH 5.5 was deposited using an inkjet printer (PIXUS iP2700, Canon). A standard setting for printing paper with high-printing quality was selected for manufacturing. The process including printing and drying at ambient temperature was repeated 10 times to obtain the solid-state fluorescent sensing sites. The effect of humidity on the PCSAD was evaluated using a thermo-hygrostat (ESPEC, SH-221).

### Chemical sensing and data acquisition

Aqueous solutions (1 μL) containing target metal ions were cast into each well of the PCSAD (4 mm^2^ per well), followed by a drying process at ambient temperature for 10 min. A smartphone (Xperia 5, Sony, Japan) with a fixed-focus lens (4.26 mm, F1.6) was employed to rapidly capture the fluorescence images of the 384-well microtiter PCSAD. The fluorescence responses of the devices were recorded under irradiation by two handy black lights (4 and 16 W at *λ*_ex_ = 365 nm) (As ONE, Japan) in a dark box. The handy black lights were set in parallel above the PCSAD to irradiate it homogeneously. The photo-capturing process was set in manual exposure mode (*i.e.*, *f*/1.4 aperture, 8 seconds shutter, and ISO of 64). The distance between the lens/black light and the PCSAD was set at 20 cm.

### Imaging analysis and pattern recognition procedures

The optical intensity data of the captured digital photographs were analyzed using an in-house-developed algorithm with MathWorks MATLAB 2023B.^11^ During the imaging analysis, the pixels related to the wax background were eliminated by a morphological detection algorithm based on the distribution of the red color channel intensity of each pixel. The imaging analysis process consisted of two steps: pixel filtering and optical intensity readout. The wells in the array were first classified into wax background or sensor pattern (*i.e.*, the detection portion) based on the red intensity of the RGB color space. After this process, the selected image fragments were transformed to color intensity data in RBG (red, blue, green), grayscale, and YC_b_C_r_ (luma component, blue- and red-differences) color spaces. The dataset was pre-processed by using the Student's *t*-test to exclude 4 outliers from the dataset with 20 repetitions for each measurement. Linear discriminant analysis (LDA)^13^ was applied for the qualitative and semi-quantitative analyses without any further treatment using SYSTAT 13. Regression analysis using a support vector machine (SVM) (*ε*-support vector regression without other data preprocessing)^42,43^ was performed for the quantitative assay with Solo 9.0. To estimate the accuracy of the established model, the root-mean-square errors of calibration (RMSEC) and prediction (RMSEP) were employed. The limit of detection (LOD) of the PCSAD was estimated by using the mean relative error values of the multisensory system.^44^

### Real-sample analysis

Real-sample analysis was demonstrated by a spike and recovery test for Ni^2+^, Cu^2+^, and Co^2+^ ions in a river water sample (Elevated Level, NMIJ CRM 7202-c) using the SVM. The pH value of the river water sample was adjusted to 5.5. The metal ions (*i.e.*, Ni^2+^ at 0.59, 0.88, and 1.17 ppm; Cu^2+^ at 0.64, 0.95, and 1.27 ppm; Co^2+^ at 0.59, 0.88, and 1.18 ppm) were spiked into the river water samples. A calibration line was built for each of Ni^2+^ (≤1.76 ppm), Cu^2+^ (≤1.91 ppm), and Co^2+^ (≤1.77 ppm) in MES buffer solution (1 mM) with NaCl (1 mM) of pH 5.5. To each well of the fabricated PCSAD, 1 μL of the target solution was dispensed using a micropipette. Owing to the low optical response in the blue color channel, only six color channels were employed for the quantitative assay to decrease the reflection of UV light.

### Morphological observation

Dynamic light scattering (DLS) measurements were performed using an ELSZ-2000 zeta-potential and particle size analyzer (Otsuka Electronics Co., Ltd). The surface morphology of the PCSAD was investigated using a Hitachi TM3030Plus tabletop microscope and a field-emission scanning electron microscope (FE-SEM, Thermo Fisher Scios 2) with energy-dispersive X-ray spectroscopy (EDX). The PCSAD samples were treated with Pd using sputtering equipment (VACUUM DEVICE, MSP-1S).

## Results and discussion

### Investigation of the optical sensing mechanism of 1_poly_

**Fig. 2:**
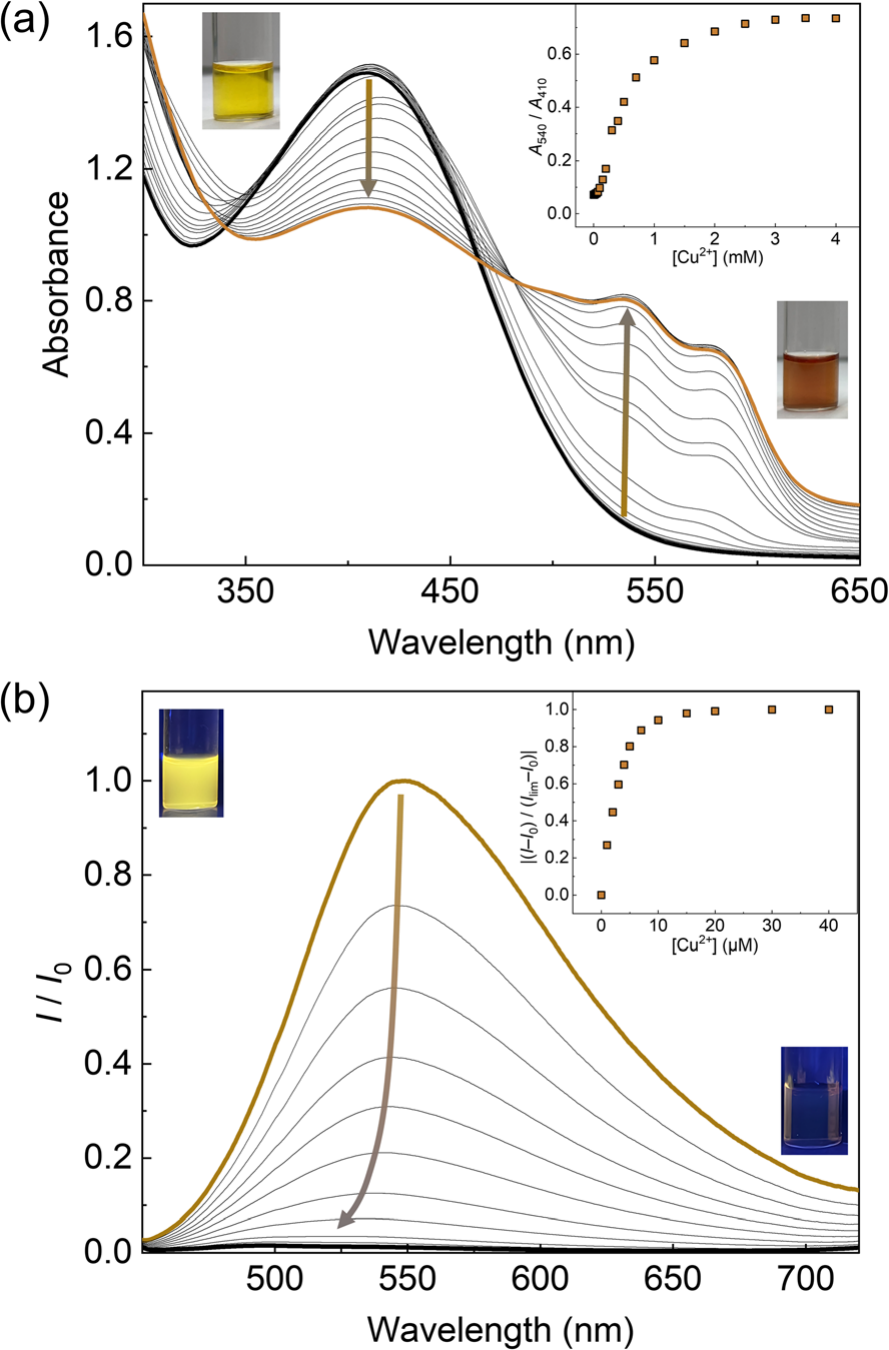
Optical sensing of Cu^2+^ ions in an aqueous MeOH solution (MeOH : water = 3 : 1, v/v) containing MES (12.5 mM) and NaCl (12.5 mM) of pH 5.5 at 25 °C. Each spectrum was recorded after mixing the 1_Poly_ solution with Cu^2+^ ions for 10 min. (a) UV-vis absorption spectra of 1_Poly_ (4.0 × 10^−4^ M per unit) upon the addition of Cu^2+^ ions (0.0–4.0 × 10^−3^ M). The insets show the concentration dependency of the relative absorbance (*A*_540_/*A*_410_) and photographs of 1_Poly_ (4.0 × 10^−4^ M per unit) (left) before and (right) after the addition of Cu^2+^ ions (4.0 × 10^−4^ M). (b) Fluorescence spectra of 1_Poly_ (4.0 × 10^−5^ M per unit) upon the addition of Cu^2+^ ions (0.0–4.0 × 10^−5^ M). *λ*_ex_ = 425 nm. The terms *I*_0_ and *I* indicate fluorescent intensity at *λ*_em_ = 550 nm before and after adding Cu^2+^ ions. The term *I*_lim_ means a saturated fluorescent response. The insets show the concentration dependency of fluorescence change and photographs of 1_Poly_ (left) before and (right) after the addition of Cu^2+^ ions under irradiation using a handy black light at 365 nm.

**Fig. 3:**
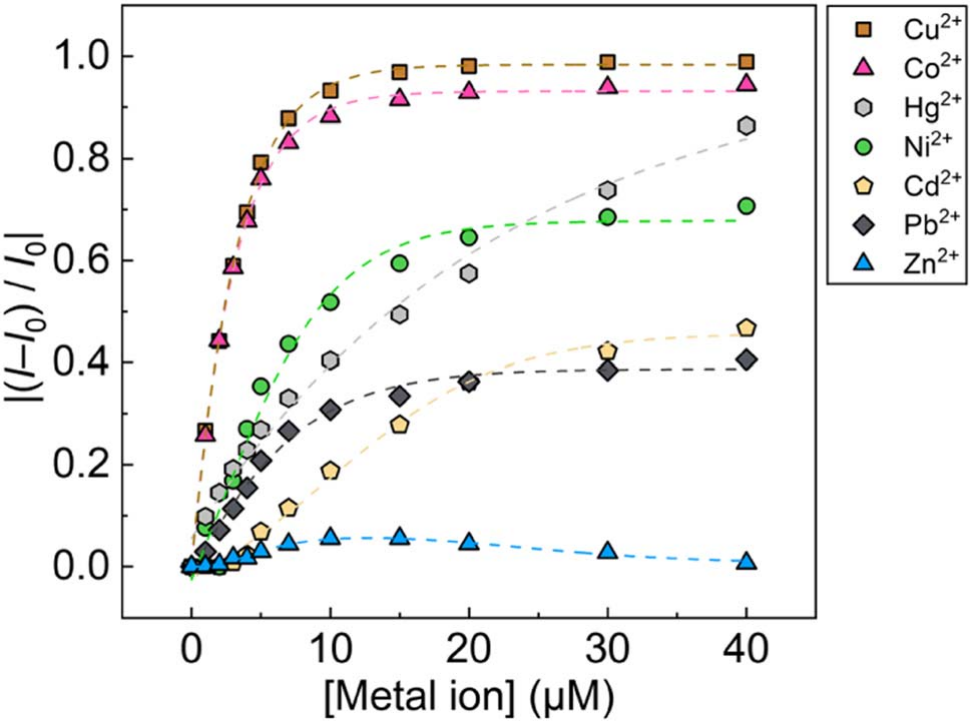
Fluorescence changes in 1_Poly_ (4.0 × 10^−5^ M per unit) upon the addition of metal ions (0.0–4.0 × 10^−5^ M) in an aqueous MeOH solution (MeOH : water = 3 : 1, v/v) containing MES (12.5 mM) and NaCl (12.5 mM) of pH 5.5 at 25 °C. The terms *I*_0_ and *I* indicate fluorescent intensity at *λ*_em_ = 550 nm before and after adding metal ions.

The optical responses of 1_Poly_ to metal ions were investigated by UV-vis and fluorescence titrations of metal ions in an aqueous MeOH solution (MeOH : water = 3 : 1, v/v) of pH 5.5 at 25 °C. Since the chemosensor 1_Poly_ showed a significant aggregation-induced response to metal ions under weak acidic conditions, pH 5.5 was selected to maximize the sensing performance of 1_Poly_ in an aqueous MeOH solution. Fig. 2(a) shows the redshift in the UV-vis absorption spectra of 1_Poly_ upon the addition of Cu^2+^ ions. The gradual redshift indicates the planarization of the thiophene backbone and further aggregation from randomly-coiled structures.^45,46^ In addition, absorption bands along with vibronic transitions were observed at 535 and 580 nm, which supported the formation of planarized structures of 1_Poly_.^41,47^ The coordination of the dpa unit modified at the sidechain of the thiophene backbone with Cu^2+^ ions was supported by the ESI-MS analysis (Fig. S1†). Moreover, DLS measurements were demonstrated to evaluate the polymer dynamics of 1_poly_ in the absence and presence of Cu^2+^ ions. The particle size of 1_poly_ was not determined in the absence of Cu^2+^ ions because of the high dispersion state of 1_poly_. On the other hand, a change in the morphology of 1_poly_ was observed in the presence of Cu^2+^ ions (Fig. S2†), which revealed that the polymer dynamics of 1_poly_ caused a redshift (Fig. 2(a)). In contrast to colorimetric changes derived from polymer dynamics in the polymer wire and/or among wires, photochemical mechanisms within the polymer wire are dominant in fluorescent chemical sensing.^28,48^ For example, Fig. 2(b) displays a significant decrease in the fluorescence intensities of 1_Poly_ with an increase in the Cu^2+^ ion concentration at μM levels. The LOD value was determined to be 16 ppb by the 3σ method.^49^ Meanwhile, although the chemosensor 1_poly_ showed a redshift in the UV-vis titration with Zn^2+^ ions (Fig. S3†), fluorescence changes were not observed (Fig. S6†). The difference in the optical behavior of 1_poly_ between metal ions can be explained by the coordination geometries causing polymer dynamics (for colorimetric responses) and energy-transfer mechanisms (for fluorescence responses). Divalent metal ions (*e.g.*, Cu^2+^, Zn^2+^, and Hg^2+^) coordinate with a dpa ligand for the formation of multiple complex modes.^37,38^ Thus, the polymer dynamics causing colorimetric changes were probably induced by the unique binding stoichiometries between the dpa ligand and metal ions, in the polymer wire and/or among wires. Furthermore, fluorescence quenchers, such as Cu^2+^, Hg^2+^, Pb^2+^, Ni^2+^, and Co^2+^ ions, cause energy transfer along the polythiophene backbone upon coordination at the Lewis-basic ligand-attached side chains.^28,48^ In this regard, polymer aggregations derived from intra- and intermolecular interactions of the polymer wires are also involved in the nonlinear quenching behavior of the polymer chemosensors.^48^ In the case of Zn^2+^ ions, a d^10^ electronic configuration does not induce energy transfer to deactivate the excited states of the fluorophores,^50^ resulting in almost no quenching of 1_Poly_ upon the addition of metal ions. The various fluorescence response patterns of 1_poly_ to metal ions (Fig. 3) provided the difference in static quenching constants (*K*_s_s),^51^ which suggested the possibility of a single polymer chemosensor for pattern recognition (Table S1†).

### Investigation of the effects of paper fibers on the dispersion of 1_poly_

**Fig. 4:**
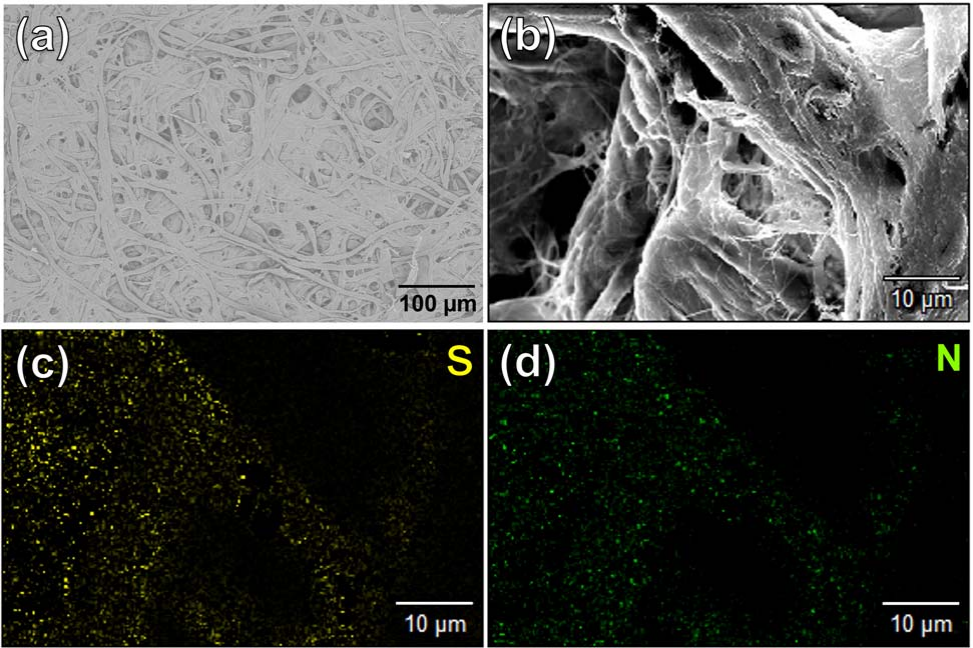
Surface morphology of the PCSAD embedded with 1_Poly_ by SEM observation with (a) 100 and (b) 1000 magnification. EDX-mapping images of (b) for (c) S and (d) N as elemental components of 1_Poly_.

**Fig. 5:**
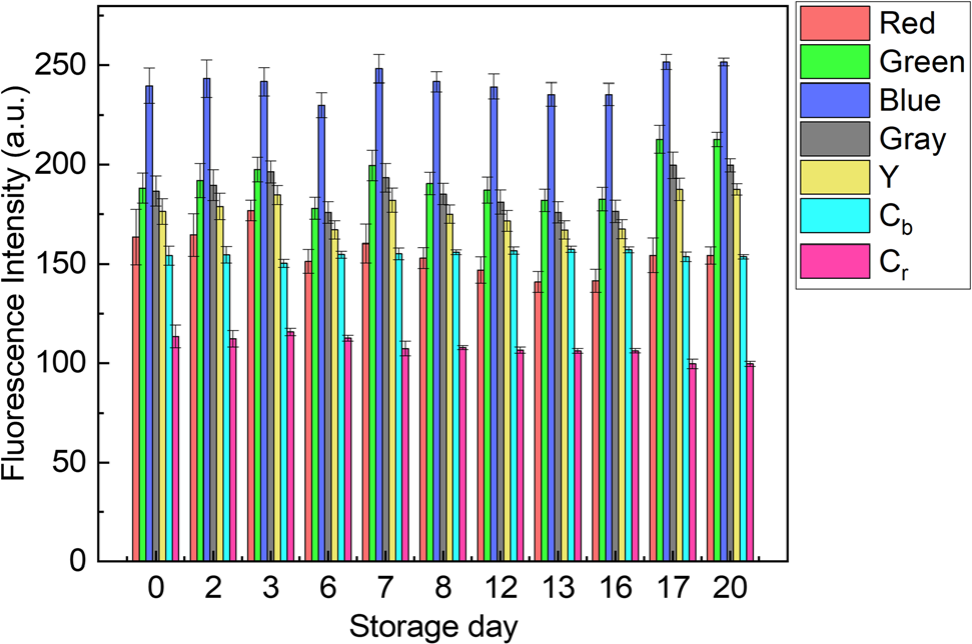
Fluorescent stability test using the 384-well microtiter PCSAD embedded with 1_Poly_. The fluorescence properties of the printed 1_Poly_ on the 384 wells were recorded at a certain time for 20 days using a smartphone. The device surface was irradiated using two handy black lights. Through the imaging analysis process, the collected digital images of the fluorescent PCSAD were visualized as a dataset with seven color channels. The PCSAD was stored at 25 °C under dark ambient conditions except for image capture.

The contribution of paper fibers to the suppression of self-aggregation quenching of 1_poly_ was evaluated by morphological observation and a fluorescent stability test in the solid state. SEM images in Fig. 4(a) and (b) show the morphology of paper fibers of the PCSAD embedded with 1_poly_ at low and high magnification, respectively. EDX mapping indicates the distribution of S and N elements contained in 1_poly_ (Fig. 4(c) and (d)). In this regard, the surface morphology of the 1_Poly_-printed PCSAD did not differ before (Fig. 4) and after adding Cu^2+^ ions (Fig. S12†). In contrast, aggregates were observed on the surface of the PCSAD after adding a drop-casting of a 1_poly_ solution containing Cu^2+^ ions (Fig. S13†). Therefore, the chemosensor 1_poly_ was highly dispersed on the paper substrate using office printing methods. Subsequently, Fig. 5 represents the average fluorescence intensity of each color channel that was extracted from 384-well microtiter PCSAD with 1_poly_ by imaging analysis. Almost no significant changes were observed in the seven color channels during the days of storage, suggesting that the aggregation-induced quenching of 1_poly_ was inhibited by the fiber structures of the paper substrate. We further evaluated the effect of humidity on the fluorescence intensities of 1_poly_ printed on the PCSAD in the thermo-hygrostat. There was almost no change in the fluorescent intensity before (15% at 25 °C) and after exposure of the device to high humidity (95% at 25 °C), indicating the robustness of this device to humidity (Fig. S14†).

### Pattern-recognition-driven chemical sensing by the PCSAD

The fluorescent sensing ability of the PCSAD was evaluated by the qualitative assay of six metal ions: Cu^2+^, Cd^2+^, Ni^2+^, Co^2+^, Pb^2+^, and Hg^2+^ ions. Upon the addition of 1 μL of the target ion solution, the PCSAD displayed various fluorescent response patterns under irradiation by two handy black lights at 365 nm (Fig. S15†). The wells embedded with 1_Poly_ exhibited fluorescence quenching patterns after the addition of 30 μM of metal ions (Fig. S16†), which closely matched the optical changes in the fluorescence titrations in solution states (Fig. 3). The LDA canonical score plot in the qualitative assay indicates the classification of six metal ions and a control sample with a 96% correction classification rate (Fig. S17†). The distance between the control cluster and the metal ion cluster in Fig. S17† corresponds to the magnitude of the fluorescence quenching of 1_Poly_ upon the addition of metal ions to the aqueous solution. In addition, although a 10 times higher concentration of 1_Poly_ was used as the chemosensor ink for the device fabrication than the concentration of 1_Poly_ for fluorescent sensing in aqueous solution, the detectable concentrations of the target metal ions were similar in solid- and the solution-state chemical sensing. Thus, the demonstration revealed the intramolecular wire effect in the solid state owing to the fiber structures of the paper substrate.

**Fig. 6:**
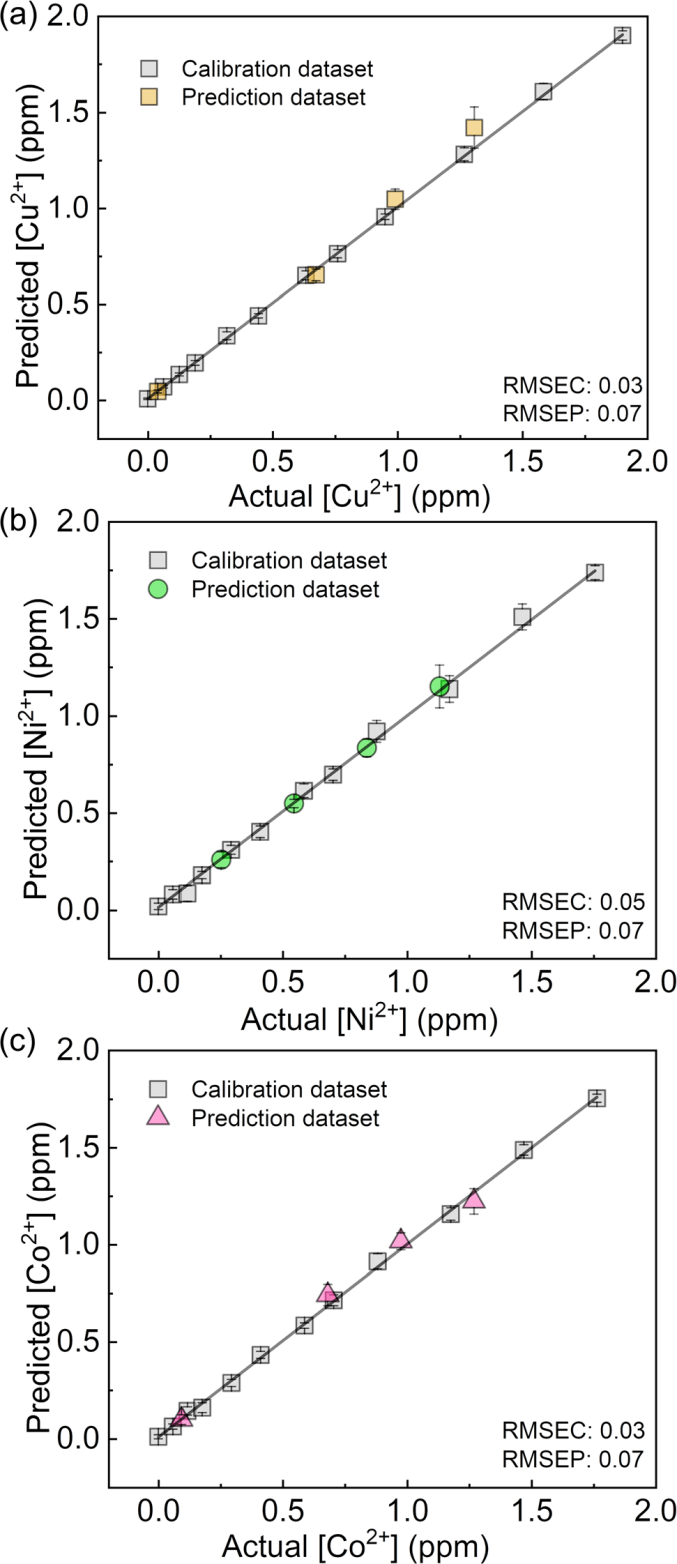
Regression analysis using SVM for mixtures of (a) Cu^2+^, (b) Ni^2+^, and (c) Co^2+^ ions in river water samples.

We performed a further semi-quantitative analysis against Cu^2+^, Co^2+^, and Ni^2+^ ions from the viewpoint of environmental assessment.^3,4^ Fig. S19† represents the concentration-dependent distribution of the metal ion clusters. Finally, the PCSAD was applied to a quantitative assay of metal ions in river water samples (Fig. 6). The SVM model contains two parts: the calibration dataset obtained from the prepared titration profiles (*i.e.*, the gray plots in Fig. 6) and the prediction dataset for the spiked metal ions (the colored plots in Fig. 6). The determined recovery rates were estimated to be 96–109% for Cu^2+^ ions, 99–102% for Ni^2+^ ions, and 96–109% for Co^2+^ ions (Table S4†). In this regard, the relatively low values of RMSEC and RMSEP indicated the high accuracy of the PCSAD. The estimated LOD of the PCSAD for Cu^2+^ ions (48 ppb) was comparable to that of the chemosensor (1_poly_) for the metal ions in the aqueous MeOH solution (16 ppb). Furthermore, we applied a quantitative assay of the Cu^2+^ ions in a commercial artificial seawater sample that is commonly used in ocean science experiments. The result of the SVM regression analysis showed the distribution of predicted concentrations of the Cu^2+^ ions on the established calibration line, indicating the applicability of the PCSAD for environmental assessments (Fig. S21†).

## Conclusions

In this study, we developed a fluorescent paper-based chemosensor array (PCSAD) using a polythiophene (PT) derivative for metal ion detection in river water. A designed and synthesized PT derivative functionalized with dipicolylamine moieties (1_poly_) showed optical changes upon the addition of target metal ions. The chemosensor 1_poly_ was printed on a paper substrate using office apparatus to disperse the PT wires homogeneously. Morphological observation by SEM suggested the effects of the fiber structures of the paper substrate on the high dispersion of 1_poly_. Indeed, a fluorescent stability test of the PCSAD indicated the role of the highly dispersed conditions of 1_poly_ for the suppression of aggregation-induced quenching. Furthermore, qualitative and quantitative detection of metal ions on the PCSAD were demonstrated using imaging analysis and pattern recognition techniques. The regression analysis of metal ions at ppm levels in river water implied the applicability of the PCSAD for real-sample analysis. From the viewpoint of sensitivity, the PCSAD-based sensing system using a smartphone (48 ppb for Cu^2+^ ions) was comparable to a solution-based sensing system using a stationary spectrophotometer (16 ppb for Cu^2+^ ions). The highly dispersed 1_Poly_ inhibited polymer dynamics, causing high factor 1 (F1) values in the LDA canonical score plots, whereas an increase in the number of chemosensors for the construction of an array can increase response spaces for pattern recognition.^52^ Overall, the sensing performance of 1_Poly_ on the paper substrate suggests that the proposed method will become a promising analytical strategy for the development of solid-state fluorescent chemosensor devices.

## Author contributions

Y. Sasaki and X. Lyu summarized the manuscript. X. Lyu and T. Kawashima performed device fabrication, solid-state chemical sensing, and data analysis. Y. Zhang demonstrated colorimetric and fluorescence titrations at solution states. The surface morphology of the PCSAD was characterized by T. Kawashima (using DLS) and K. Ohshiro (using SEM). ESI-MS analysis was performed by K. Okabe. The polythiophene derivative was synthesized by K. Tsuchiya. The entire project was conceived by T. Minami.

## Conflicts of interest

There are no conflicts of interest to declare.

## Supplementary Material

RA-014-D3RA08429A-s001
