# Supplementary material for: Nanoarchitectonics of highly dispersed polythiophene on paper for accurate quantitative detection of metal ions
Source: RSC Adv. 2024 Feb 8;14(8):5159–66. doi: 10.1039/d3ra08429a (PMC10851342; doi:10.1039/d3ra08429a)
Supplement: RA-014-D3RA08429A-s001 [file RA-014-D3RA08429A-s001.pdf]

Electronic Supplementary Material (ESI) for RSC Advances.
This journal is © The Royal Society of Chemistry 2024

Electronic Supplementary Information

Nanoarchitectonics of highly dispersed polythiophene on paper for accurate quantitative detection of metal ions

Yui Sasaki,[a,b] Xiaojun Lyu,[a] Takayuki Kawashima,[a] Yijing Zhang,[a] Kohei Ohshiro,[a] Kiyosumi Okabe,[a] Kazuhiko Tsuchiya[a] and Tsuyoshi Minami*[a]

[a.] Institute of Industrial Science, The University of Tokyo, 4-6-1 Komaba, Meguro-ku, Tokyo, 153-8505, Japan. E-mail: tminami@g.ecc.u-tokyo.ac.jp

[b.] JST, PRESTO, 4-1-8 Honcho, Kawaguchi, Saitama, 332-0012, Japan.

Contents

1. Synthesis of $\mathbf{1}_{\mathbf{poly}}$ S2
2. ESI-MS analysis S2
3. DLS measurement S2
4. Selected UV-vis titrations S3
5. Selected fluorescence titrations S4
6. Static quenching constants S6
7. Morphology analysis S7
8. Humidity dependency S7
9. Qualitative assay using LDA S8
10. Semi-quantitative using by LDA S10
11. Real-sample analysis using SVM S11

References S13

1. Synthesis of 1_{poly}

The polythiophene derivative (**1**$_{poly}$) was obtained by oxidative polymerization of a dpa-attached thiophene monomer (**1**) with $FeCl_3$.[S1] Monomer **2** was synthesized according to a previous report, [S2] for obtaining **1** by the introduction of 2,2'-dipicolylamine into **2**. A methyl group-substituted thiophene monomer at 4-position was employed to avoid the generation of byproducts (*α-β'* coupling) in the polymerization process.[S3,S4] The polymerization method was selected from the viewpoint of reactivity to heterocyclic amine-attached thiophene monomers and the yield of homopolymers.[S5,S6] The polymerization was carried out after substituting the dpa unit into the thiophene ring to avoid the formation of heterogeneous copolymers of **2** and unreacted starting material (**1**). In addition, thermal extraction was performed with a mixture of methanol and hydrazine monohydrate to remove Fe ions from the obtained product. NMR analysis result of **1**$_{poly}$ indicated that regioselective product with head-to-tail orientation with high yields (80–90%).[S1,S6]

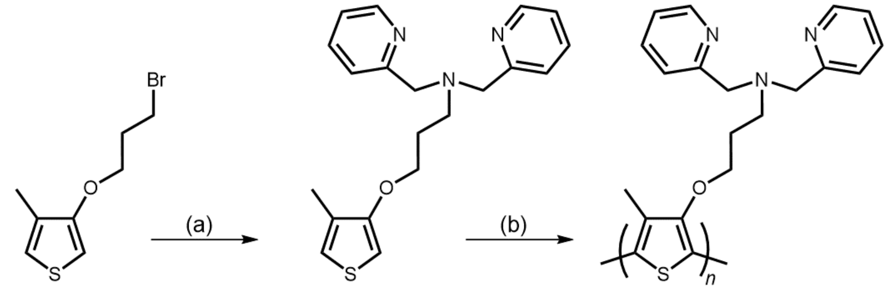

2 1 1_{poly}

Scheme S1 Synthesis of the dpa-attached PT (**1**$_{poly}$). (a) 2,2'-Dipicolylamine, K_2CO_3, CH_3CN, reflux, (b) $FeCl_3$, dry $CHCl_3$, r. t. These materials, **1**, **2**, and **1**$_{poly}$ were obtained according to a previously reported protocol.[S1]

2. ESI-MS analysis

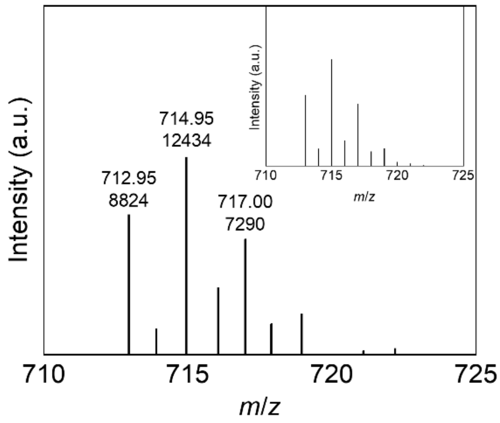

Fig. S1 An ESI-MS spectrum for the complex of **1** and $Cu(ClO_4)_2$ in MeOH. The inset shows calculated isotope patterns. [**1**] = [$Cu(ClO_4)_2$] = 2.8×10^{-3} M. Mobile phase: MeOH. MS (ESI, –) *m/z*: [**1**+Cu+3ClO_4]$^-$, $C_{20}H_{23}Cl_3CuN_3O_{13}S$ 715.36; Found: 714.95.

3. DLS measurement

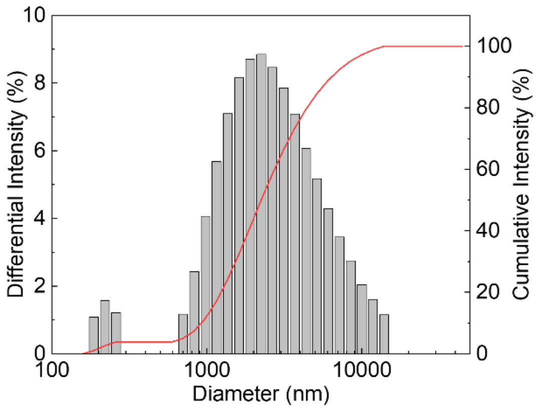

Fig. S2 Histogram for the particle-size distribution of **1**$_{\mathbf{poly}}$ (4.0×10^{-4} M/unit) upon the addition of Cu^{2+} ions (3.0 ×10^{-3} M) in an aqueous MeOH solution (MeOH:water = 3:1, v/v) containing MES (12.5 mM) and NaCl (12.5 mM) at pH 5.5 at 25 °C.

4. Selected UV-vis titrations

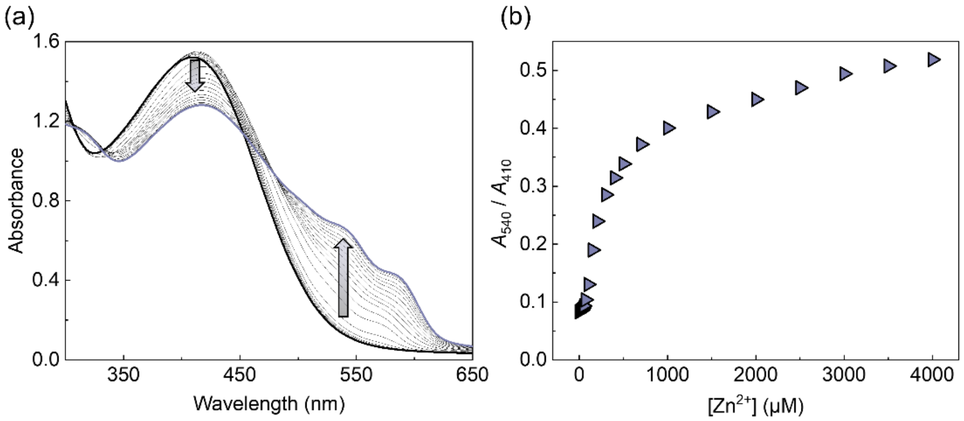

Fig. S3 (a) UV-vis absorption spectra of **1**$_{\mathbf{Poly}}$ (4.0×10^{-4} M/unit) upon the addition of Zn^{2+} ions in an aqueous MeOH solution (MeOH:water = 3:1, v/v) containing MES (12.5 mM) and NaCl (12.5 mM) at pH 5.5 at 25 °C. (b) Concentration dependency of the relative absorbance (A_{540}/A_{410}). [Zn^{2+}] = 0.0 – 4.0×10^{-3} M.

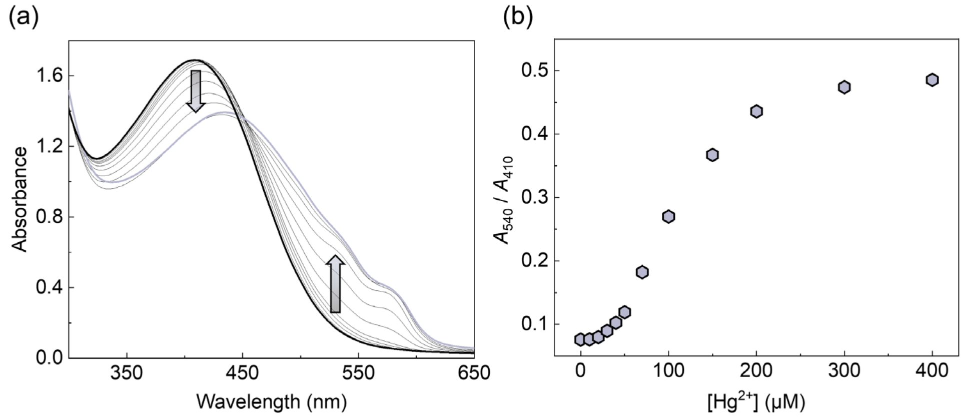

Fig. S4 (a) UV-vis absorption spectra of **1**$_{\mathbf{Poly}}$ (4.0×10^{-4} M/unit) upon the addition of Hg^{2+} ions in an aqueous MeOH solution (MeOH:water = 3:1, v/v) containing MES (12.5 mM) and NaCl (12.5 mM) at pH 5.5 at 25 °C. (b) Concentration dependency of the relative absorbance (A_{540}/A_{410}). [Hg^{2+}] = 0.0 – 4.0×10^{-4} M.

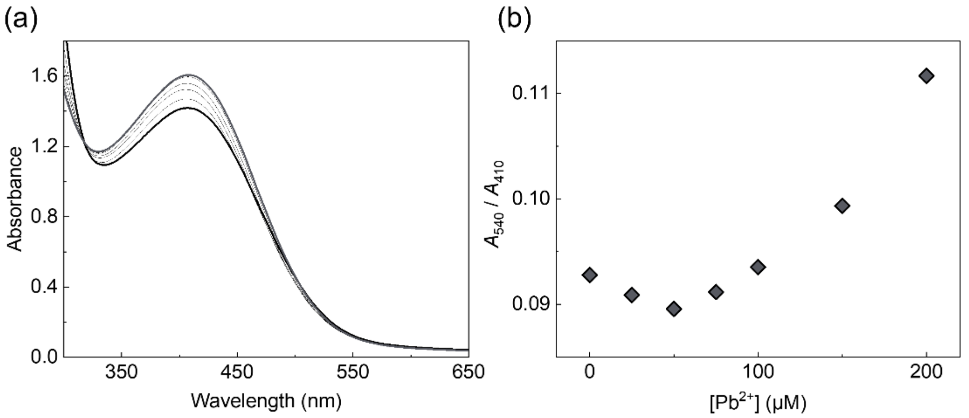

Fig. S5 (a) UV-vis absorption spectra of $\mathbf{1}_{\mathbf{Poly}}$ (4.0×10^{-4} M/unit) upon the addition of Pb^{2+} ions in an aqueous MeOH solution (MeOH:water = 3:1, v/v) containing MES (12.5 mM) and NaCl (12.5 mM) at pH 5.5 at 25 °C. (b) Concentration dependency of the relative absorbance (A_{540}/A_{410}). $[Pb^{2+}] = 0.0-2.0\times10^{-4}$ M. Precipitation was observed at high concentrations of the Pb^{2+} ions ($\geq 3.0\times10^{-4}$ M).

5. Selected fluorescence titrations

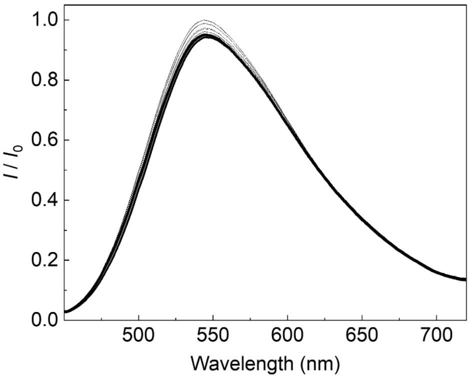

Fig. S6 Fluorescence spectra of $\mathbf{1}_{\mathbf{Poly}}$ (4.0×10^{-5} M/unit) upon the addition of Zn^{2+} ions in an aqueous MeOH solution (MeOH:water = 3:1, v/v) containing MES (12.5 mM) and NaCl (12.5 mM) at pH 5.5 at 25 °C. λ_{ex} = 425 nm. $[Zn^{2+}] = 0.0-4.0\times10^{-5}$ M.

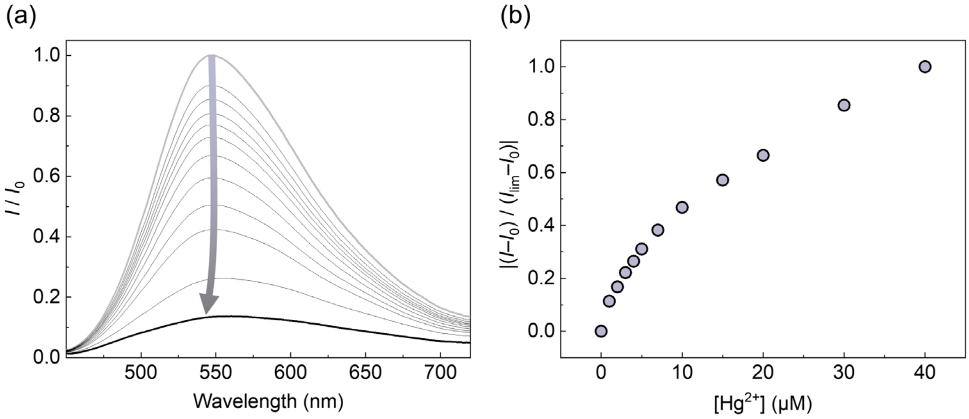

Fig. S7 (a) Fluorescence spectra of $\mathbf{1}_{\mathbf{Poly}}$ (4.0×10^{-5} M/unit) upon the addition of Hg^{2+} ions in an aqueous MeOH solution (MeOH:water = 3:1, v/v) containing MES (12.5 mM) and NaCl (12.5 mM) at pH 5.5 at 25 °C. λ_{ex} = 425 nm. (b) Concentration dependency of fluorescence change at λ_{em} = 550 nm. $[Hg^{2+}] = 0.0-4.0\times10^{-5}$ M.

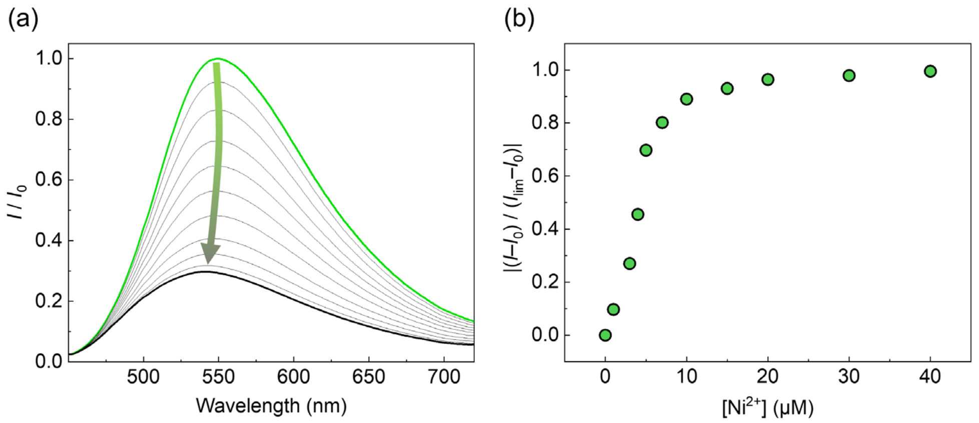

Fig. S8 (a) Fluorescence spectra of **1**$_{\mathbf{Poly}}$ (4.0×10^{-5} M/unit) upon the addition of Ni^{2+} ions in an aqueous MeOH solution (MeOH:water = 3:1, v/v) containing MES (12.5 mM) and NaCl (12.5 mM) at pH 5.5 at 25 °C. λ_{ex} = 425 nm. (b) Concentration dependency of fluorescence change at λ_{em} = 550 nm. [Ni^{2+}] = 0.0 − 4.0×10^{-5} M.

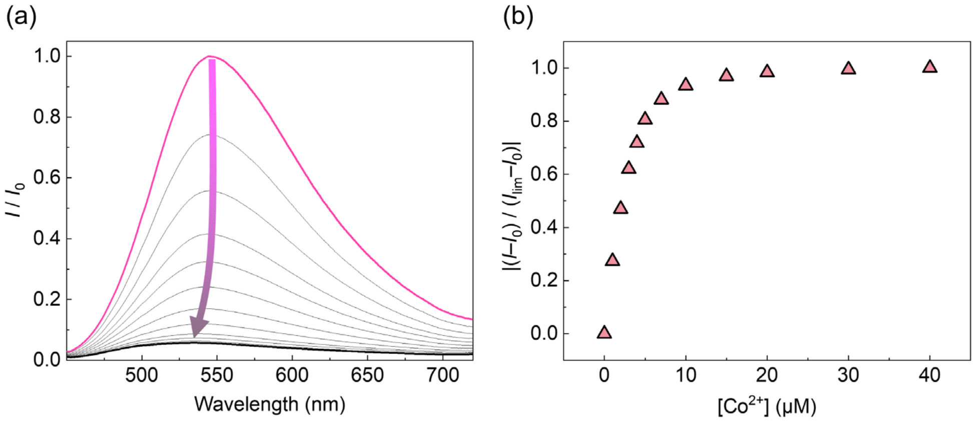

Fig. S9 (a) Fluorescence spectra of **1**$_{\mathbf{Poly}}$ (4.0×10^{-5} M/unit) upon the addition of Co^{2+} ions in an aqueous MeOH solution (MeOH:water = 3:1, v/v) containing MES (12.5 mM) and NaCl (12.5 mM) at pH 5.5 at 25 °C. λ_{ex} = 425 nm. (b) Concentration dependency of fluorescence change at λ_{em} = 550 nm. [Co^{2+}] = 0.0 − 4.0×10^{-5} M.

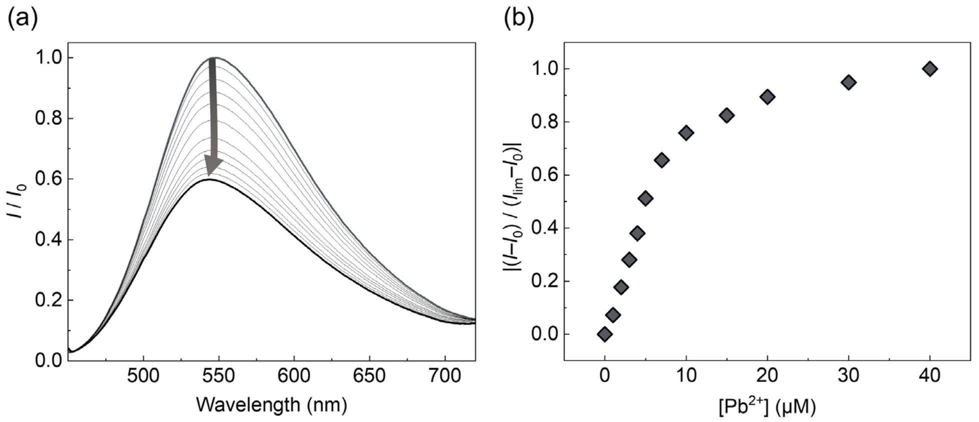

Fig. S10 (a) Fluorescence spectra of **1**$_{\mathbf{Poly}}$ (4.0×10^{-5} M/unit) upon the addition of Pb^{2+} ions in an aqueous MeOH solution (MeOH:water = 3:1, v/v) containing MES (12.5 mM) and NaCl (12.5 mM) at pH 5.5 at 25 °C. λ_{ex} = 425 nm. (b) Concentration dependency of fluorescence change at λ_{em} = 550 nm. [Pb^{2+}] = 0.0 − 4.0×10^{-5} M.

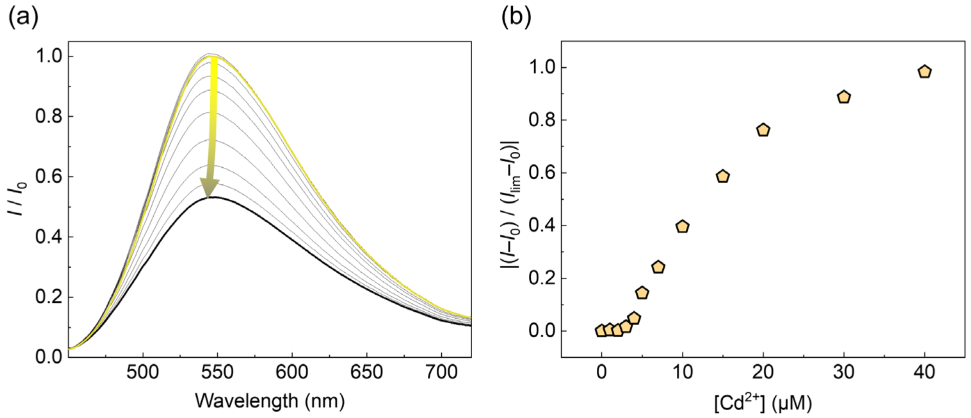

Fig. S11 (a) Fluorescence spectra of **1**$_{\mathbf{Poly}}$ (4.0×10^{-5} M/unit) upon the addition of Cd^{2+} ions in an aqueous MeOH solution (MeOH:water = 3:1, v/v) containing MES (12.5 mM) and NaCl (12.5 mM) at pH 5.5 at 25 °C. λ_{ex} = 425 nm. (b) Concentration dependency of fluorescence change at λ_{em} = 550 nm. [Cd^{2+}] = 0.0 — 4.0×10^{-5} M.

6. Static quenching constants

With an increase in quencher concentrations (*i.e.*, metal ions), the decrease in fluorescence emission intensity of the fluorophore (**1**$_{\mathbf{poly}}$) corresponds to the quencher concentrations as described by the Stern–Volmer relationship (eq. 1);[S7]

$$\frac{I_0}{I} = (1 + K_{SV}[Q])(1 + K_S[Q]) = 1 + (K_{SV} + K_S)[Q] + K_{SV}K_S[Q]^2 \qquad (1)$$

where I_0 and I are the fluorescence emission intensity in the absence and the presence of a quencher metal ion, K_{SV} is the Stern-Volmer quenching constant (for dynamic quenching), K_S is the static quenching constant, and [Q] is the concentration of the quencher metal ions. Since K_S is more dominant than K_{SV} in the case of polymer-based fluorescent materials in aqueous buffer solutions, the dynamic quenching is ignorable.[S8]

Table S1 The static quenching constants of **1**$_{\mathbf{Poly}}$ (M^{-1})

Metal ions	K_S (M^{-1})
Zn^{2+}	N.D.[a)]
Cu^{2+}	2.0×10^6
Cd^{2+}	2.6×10^4
Ni^{2+}	1.2×10^5
Co^{2+}	8.2×10^5
Pb^{2+}	4.6×10^4
Hg^{2+}	7.0×10^3

a) N.D.: not determined owing to no contribution as the quencher metal ion.

7. Morphology analysis

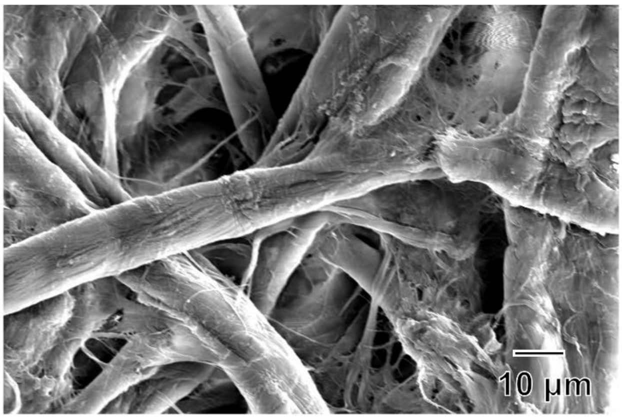

Fig. S12 FE-SEM image of the $\mathbf{1}_{\mathbf{Poly}}$-printed PCSAD after adding Cu^{2+} ions (3.0 $\times10^{-3}$ M).

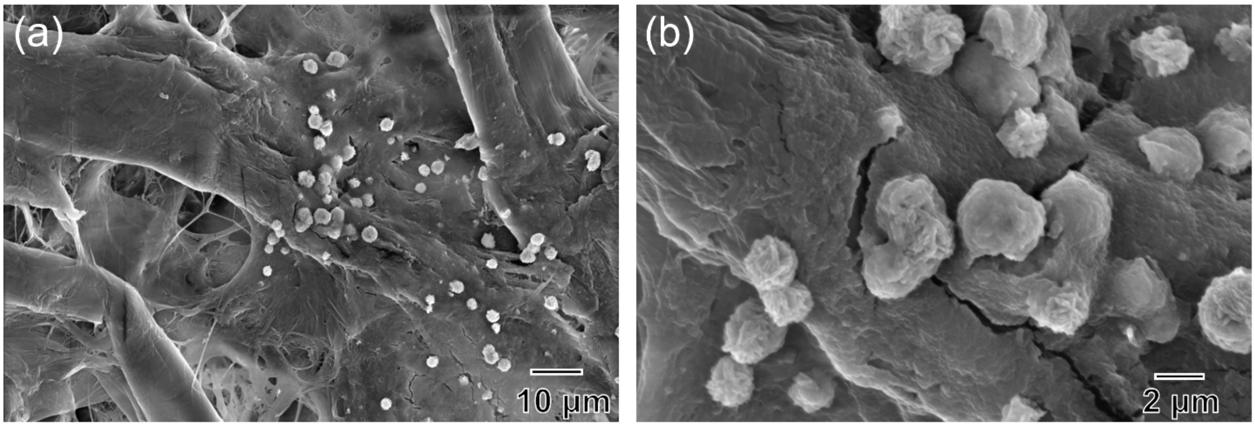

Fig. S13 FE-SEM images of the PCSAD after drop-casting of a mixture of $\mathbf{1}_{\mathbf{Poly}}$ (4.0$\times10^{-4}$ M/unit) and Cu^{2+} ions (3.0 $\times10^{-3}$ M) at (a) 1000 and (b) 5000 magnification.

8. Humidity dependency

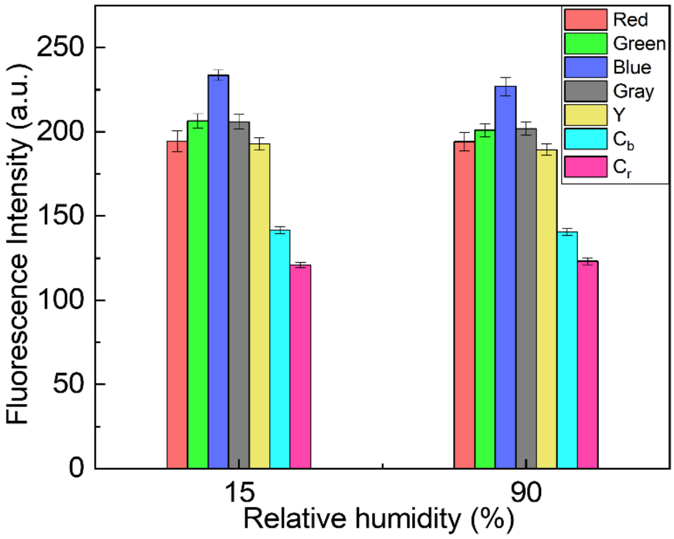

Fig. S14 The fluorescence intensity of the printed $\mathbf{1}_{\mathbf{Poly}}$ on the 384 wells before (15% at 25 °C) and after exposure to high humidity (95% at 25 °C).

Qualitative assay using LDA

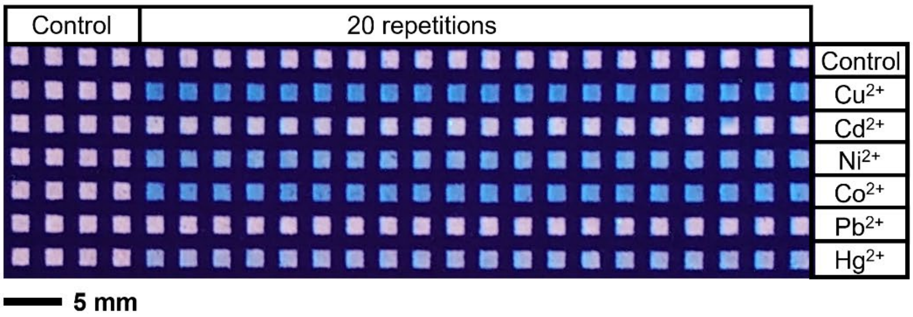

Fig. S15 Fluorescent responses of the PCSAD embedded with $\mathbf{1}_{\mathbf{poly}}$ upon the addition of metal ions (5.0×10^{-5} M), which was acquired by using a smartphone. The surface of the PCSAD was irradiated by using two handy black lights (4 and 16 W at λ_{ex} = 365 nm).

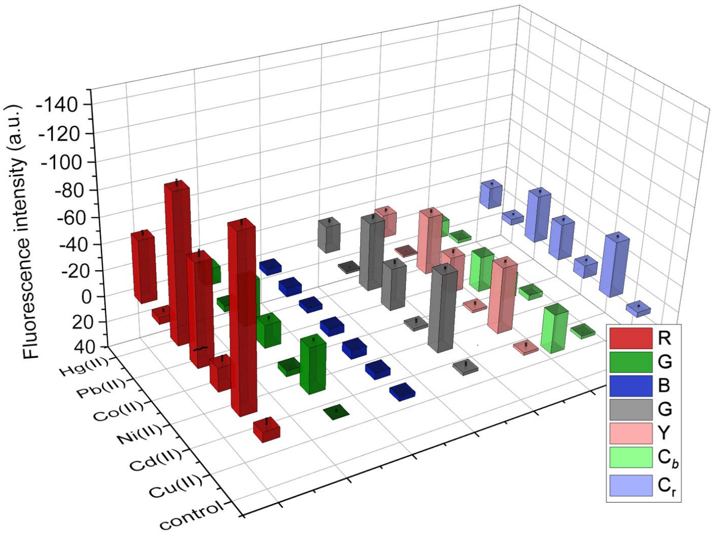

Fig. S16 Fluorescent intensity profile on the 384-well microtiter PCSAD upon the addition of metal ions in an MES buffer solution (1 mM) with NaCl (1 mM) at pH 5.5. The fluorescence responses of the devices after adding metal ions (1 µL/well, 3.0×10^{-5} M) were recorded under the irradiation of two handy black lights at 365 nm. Δ Fluorescence intensity before and after adding metal ions was employed for the bar graph.

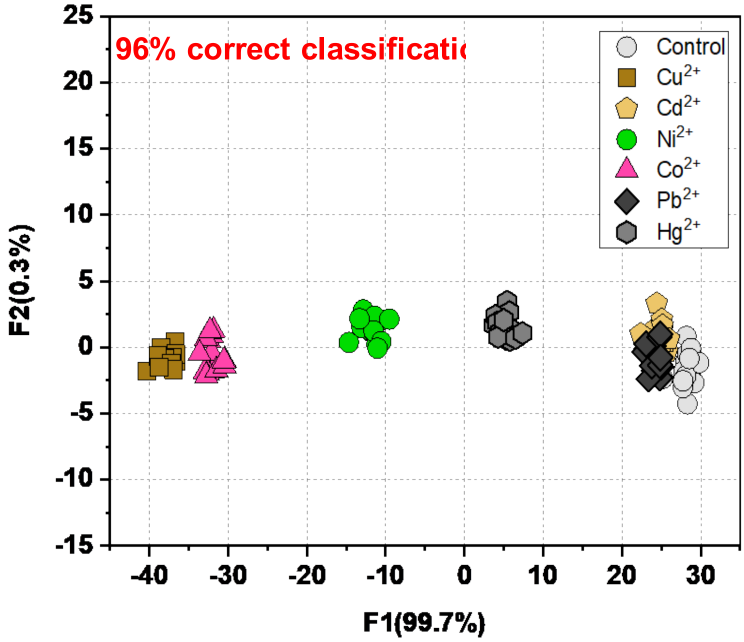

Fig. S17 Qualitative assay against 6 metal ions (3.0×10^{-5} M). Each measurement was repeated 16 times.

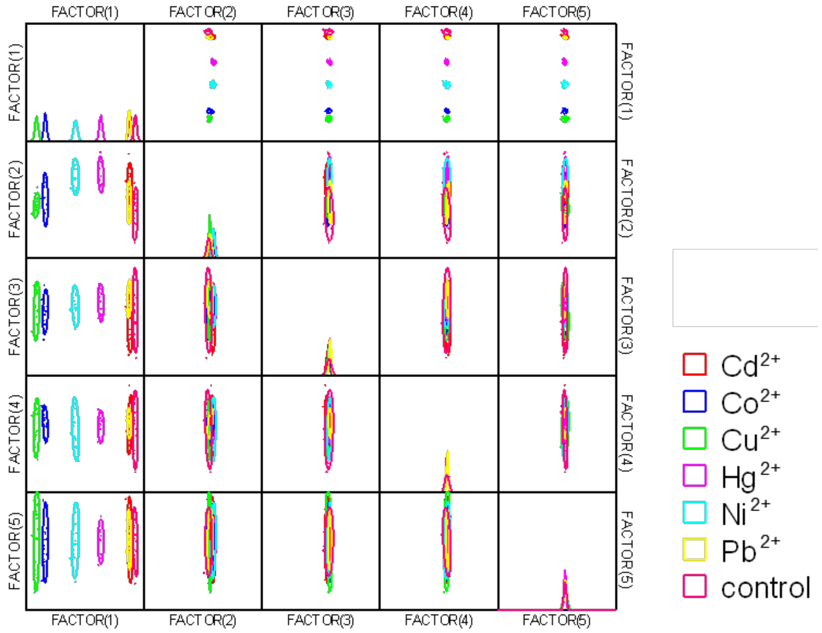

Fig. S18 The canonical score plot of the qualitative assay.

Table S2 The jackknifed classification matrix of the qualitative assay for 6 metal ions

	Cd^{2+}	Co^{2+}	Cu^{2+}	Hg^{2+}	Ni^{2+}	Pb^{2+}	control	%correct
Cd^{2+}	13	0	0	0	0	3	0	81
Co^{2+}	0	16	0	0	0	0	0	100
Cu^{2+}	0	0	16	0	0	0	0	100
Hg^{2+}	0	0	0	16	0	0	0	100
Ni^{2+}	0	0	0	0	16	0	0	100
Pb^{2+}	1	0	0	0	0	15	0	94
control	0	0	0	0	0	1	15	94
Total	14	16	16	16	16	19	15	96

9. Semi-quantitative assay using LDA

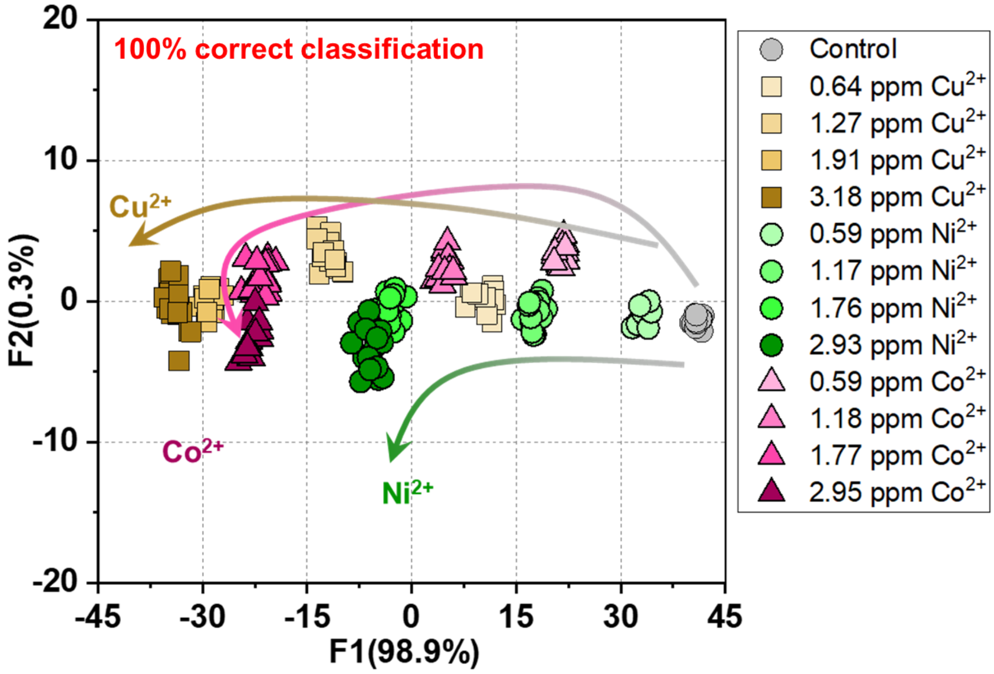

Fig. S19 Semi-quantitative assay against Cu^{2+}, Ni^{2+}, and Co^{2+} ions. Each measurement was repeated 16 times.

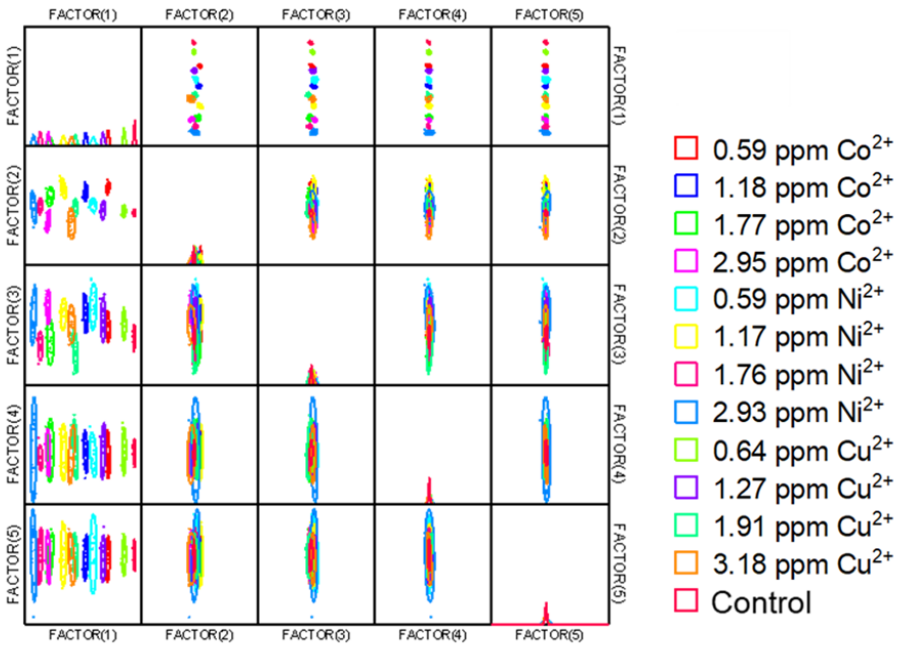

Fig. S20 The canonical score plot of the semi-quantitative assay.

Table S3 The jackknifed classification matrix of the semi-quantitative assay for Co^{2+}, Ni^{2+}, and Cu^{2+} ions

	Co^{2+} 0.59 ppm	Co^{2+} 1.18 ppm	Co^{2+} 1.77 ppm	Co^{2+} 2.95 ppm	Co^{2+} 0.59 ppm	Ni^{2+} 0.59 ppm	Ni^{2+} 1.17 ppm	Ni^{2+} 1.76 ppm	Ni^{2+} 2.93 ppm	Cu^{2+} 0.64 ppm	Cu^{2+} 1.27 ppm	Cu^{2+} 1.91 ppm	Cu^{2+} 3.18 ppm	control	%correct
Co^{2+} 0.59 ppm	16	0	0	0	0	0	0	0	0	0	0	0	0	0	100
Co^{2+} 1.18 ppm	0	16	0	0	0	0	0	0	0	0	0	0	0	0	100
Co^{2+} 1.77 ppm	0	0	16	0	0	0	0	0	0	0	0	0	0	0	100
Co^{2+} 2.95 ppm	0	0	0	16	0	0	0	0	0	0	0	0	0	0	100
Co^{2+} 0.59 ppm	0	0	0	0	16	0	0	0	0	0	0	0	0	0	100
Ni^{2+} 0.59 ppm	0	0	0	0	0	16	0	0	0	0	0	0	0	0	100
Ni^{2+} 1.17 ppm	0	0	0	0	0	0	16	0	0	0	0	0	0	0	100
Ni^{2+} 1.76 ppm	0	0	0	0	0	0	0	16	0	0	0	0	0	0	100
Ni^{2+} 2.93 ppm	0	0	0	0	0	0	0	1	15	0	0	0	0	0	100
Cu^{2+} 0.64 ppm	0	0	0	0	0	0	0	0	0	16	0	0	0	0	94
Cu^{2+} 1.27 ppm	0	0	0	0	0	0	0	0	0	0	16	0	0	0	100
Cu^{2+} 1.91 ppm	0	0	0	0	0	0	0	0	0	0	0	16	0	0	100
Cu^{2+} 3.18 ppm	0	0	0	0	0	0	0	0	0	0	0	0	16	0	100
control	0	0	0	0	0	0	0	0	0	0	0	0	0	16	100
Total	16	16	16	16	16	16	16	17	15	16	16	16	16	16	100

10.Real-sample analysis using SVM

Table S4 The spiked and recovery test using the PCSAD for metal ions in the river water sample

		Added (ppm)	Found (ppm)	Recovery (%)		Added (ppm)	Found (ppm)	Recovery (%)		Added (ppm)	Found (ppm)	Recovery (%)
**River water**	Cu^{2+}	0.64	0.61	96	Co^{2+}	0.59	0.64	109	Ni^{2+}	0.29	0.29	99
		0.95	1.00	105		0.88	0.92	104		0.59	0.58	99
		1.27	1.38	109		1.18	1.13	96		0.88	0.90	102

Table S5 The elements of the original river water sample

Element	Concentration
B	44.0 μg/kg
Al	21.8 μg/kg
Cr	5.16 μg/kg
Mn	5.04 μg/kg

Fe	27.1μg/kg
Ni	1.06 μg/kg
Cu	10.1 μg/kg
Zn	10.6 μg/kg
As	1.17 μg/kg
Se	1.03 μg/kg
Rb	0.653 μg/kg
Sr	33.5 μg/kg
Mo	0.183 μg/kg
Cd	1.01 μg/kg
Sb	0.0095 μg/kg
Ba	5.74 μg/kg
Pb	1.018 μg/kg
Na	3.68 mg/kg
Mg	1.26 mg/kg
K	0.836 mg/kg
Ca	4.59 mg/kg

Table S6. The spiked and recovery test for the Cu^{2+} ions in the commercial artificial seawater sample

		**Added (ppm)**	**Found (ppm)**	**Recovery (%)**
**Commercial artificial seawater sample**	**Cu^{2+}**	0.64	0.56	88%
		0.95	0.78	81%
		1.27	1.07	84%

Table S7. The ingredient list of the commercial artificial seawater sample

**Ingredient**	**Concentration (ppm)**
$MgCl_2 \cdot 6H_2O$	9474
$CaCl_2 \cdot 2H_2O$	1326
Na_2SO_4	3505
KCl	597
$NaHCO_3$	171
KBr	85
$Na_2B_4O_7 \cdot 10H_2O$	34
$SrCl_2$	12
NaF	3
LiCl	1

KI	0.07
$CoCl_2 \cdot 6H_2O$	0.0002
$AlCl_3 \cdot 6H_2O$	0.008
$FeCl_3 \cdot 6H_2O$	0.005
$Na_2WO_4 \cdot 2H_2O$	0.0002
$(NH_4)_6Mo_7O_{24} \cdot 4H_2O$	0.02
$MnCl_2 \cdot 4H_2O$	0.0008
NaCl	20747

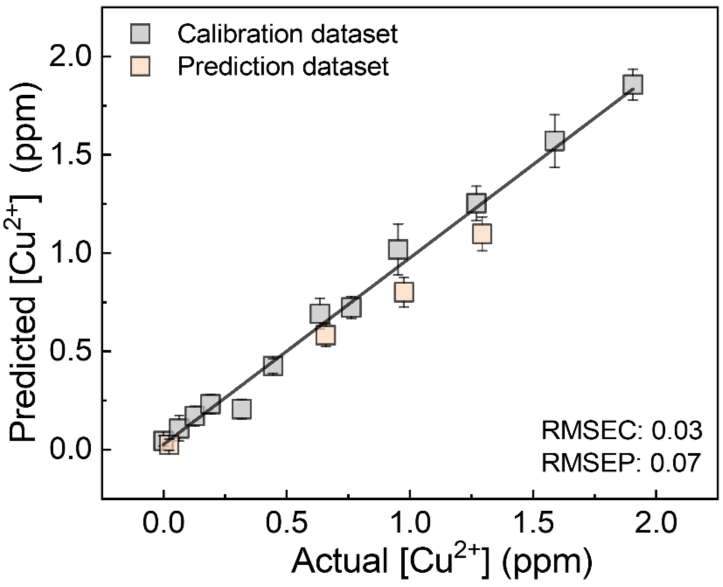

Fig. S21 Regression analysis using SVM for the Cu^{2+} ions in the commercial artificial seawater sample.

References

1. Y. Sasaki, K. Ohshiro, K. Okabe, X. Lyu, K. Tsuchiya, A. Matsumoto, S. Takizawa and T. Minami, *Chem. Asian. J.*, 2023, **18**, e202300372.
2. D. Cheng, Y. Li, J. Wang, Y. Sun, L. Jin, C. Li and Y. Lu, *Chem. Commun*. 2015, **51**, 8544.
3. K. Tsuchiya and K. Ogino, *Polym. J.* 2013, **45**, 281.
4. D. Thanasamy, D. Jesuraj, S. K. Konda Kannan and V. Avadhanam, *Polymer* 2019, **175**, 32.
5. K. Yao, L. Chen, F. Li, P. Wang and Y. Chen, *J. Phys. Chem. C* 2012, **116**, 714.
6. S. Amou, O. Haba, K. Shirato, T. Hayakawa, M. Ueda, K. Takeuchi and M. Asai, *J. Polym. Sci.* 1999, **37**, 1943.
7. L. Cisse, A. Djande, M. Capo-Chichi, F. Delattre, A. Saba, J.-C. Brochon, S. Sanouski, A. Tine and J.-J. Aaron, *J. Fluoresc.*, 2017, **27**, 619-628.
8. J. Wang, D. Wang, E. K. Miller, D. Moses, G. C. Bazan, and A. J. Heeger, *Macromolecules*, 2000, **33**, 5153–5158.
